# Optimizing preoperative assessment of salivary gland lesions: updates and practical implications in the Milan System 2nd edition

**DOI:** 10.1007/s00428-026-04508-z

**Published:** 2026-04-08

**Authors:** Patrizia Straccia, Vincenzo Fiorentino, Alessia Piermattei, Qianqian Zhang, Belen Padial-Urtueta, Federica Cianfrini, Antonino Mule’, Esther Diana Rossi

**Affiliations:** 1 Division of Anatomic Pathology and Histology, Fondazione Policlinico Universitario “Agostino Gemelli”-IRCCS, Università Cattolica del Sacro Cuore, Largo Francesco Vito, 1, 00168 Rome, Italy; 2 Anatomic Pathology Unit, Department of Human Pathology in Adult and Developmental Age “Gaetano Barresi”, University of Messina, Messina, Italy

**Keywords:** Salivary gland, Fine-needle aspiration, Milan System 2nd edition, Cell-block, ROSE, Telecytology, Immunohistochemistry, Molecular diagnostics, Risk of malignancy

## Abstract

Salivary gland lesions encompass a broad clinicopathologic spectrum and remain challenging in the preoperative setting because tumor heterogeneity, cystic change, and cytomorphologic overlap may limit confident classification on fine-needle aspiration alone. The Milan System for Reporting Salivary Gland Cytopathology has standardized reporting by linking diagnostic categories to risk of malignancy and clinical management, while its second edition places greater emphasis on integration with imaging and ancillary testing. This narrative review discusses how these updated principles can be translated into daily practice through rapid on-site evaluation or telecytology, cell-block preparation, focused immunohistochemical and molecular studies, and selective use of ultrasound-guided core needle biopsy. The available evidence indicates that cell blocks improve diagnostic yield and facilitate ancillary testing, whereas non-contributory cell blocks should alert the pathologist to possible suboptimal sampling. Comparative studies and meta-analyses further show that core needle biopsy yields higher sensitivity and lower non-diagnostic rates than fine-needle aspiration, with uncommon and usually minor complications. Overall, an integrated, imaging-informed preoperative pathway can reduce indeterminate reporting, increase specific diagnoses, and support more timely and clinically actionable management of salivary gland lesions.

## Introduction

Salivary gland pathology sits at the intersection of rarity and heterogeneity. Salivary gland malignancies are rare, representing roughly 1–5% of head and neck cancers. Most salivary gland nodules arise in the major glands (particularly the parotid) and are benign, with pleomorphic adenoma and Warthin tumor among the most common entities [1]. Although most salivary gland nodules are benign, malignant tumors are clinically consequential because operative planning may hinge on anticipating aggressive behavior, facial nerve risk, nodal evaluation, and adjuvant therapy needs [2, 3]. Preoperative sampling is expected to answer two management-defining questions: whether a lesion is non-neoplastic versus neoplastic (often determining conservative management versus surgery) and, among neoplasms, whether it is benign versus malignant (influencing the extent of surgery and, in parotid lesions, facial nerve–sparing strategies). Preoperative sampling therefore carries a disproportionate responsibility: it must triage patients efficiently while providing information sufficiently robust to guide real-world decisions. Fine-needle aspiration (FNA) has long been central to this effort because it is minimally invasive, rapid, and inexpensive, and because it can spare surgery in non-neoplastic conditions while directing neoplasms toward appropriate intervention [4, 5].

At the same time, salivary cytology has intrinsic vulnerabilities. Tumor heterogeneity is common; cystic change can dilute diagnostic material; and distinct entities may share similar cytomorphologic patterns, limiting confident subtyping or grading on smears alone [4, 6]. Across series, FNA performance is variable (reported sensitivities ~ 80–92% and specificities ~ 93–100%), reflecting the intrinsic sampling limitation and strong dependence on targeting technique, specimen quality, interpretive expertise, and cystic components [4, 7]. This is precisely why the field has moved toward both standardized reporting and workflow-driven optimization. The Milan System for Reporting Salivary Gland Cytopathology (MSRSGC) provides a shared language by linking categories to risk of malignancy (ROM) and management recommendations [6, 8]. Its second edition (Milan System 2nd edition), published in July 2023, reinforces a contemporary expectation: salivary cytology is not simply “slide interpretation,” but a node within an integrated diagnostic pathway that explicitly incorporates imaging context and ancillary testing [6, 8].

In this review, we focus on how to translate Milan System 2nd edition into routine practice by combining rapid adequacy assessment (rapid on-site evaluation (ROSE)/tele-ROSE), systematic or strategically deployed cell-block preparation, and selective escalation to core needle biopsy (CNB), while embedding ancillary immunohistochemistry (IHC)/fluorescence in situ hybridization (FISH)/next-generation sequencing (NGS) into a disciplined triage strategy.

This review is intended to complement previous Milan System 2nd edition-focused literature [6, 8]. Its added value is a workflow-level perspective that integrates imaging-guided sampling, ROSE/tele-ROSE, cell-block triage, focused ancillary testing, and selective escalation to CNB into a single preoperative pathway, with particular attention to how gray-zone scenarios can be managed in daily practice and how ROM should be interpreted when different studies use different follow-up denominators.

## Scope and literature selection

This article was conceived as a narrative, practice-oriented review rather than a formal systematic review. PubMed-indexed, peer-reviewed English-language publications available through February 2026 were prioritized using combinations of the terms salivary gland, fine-needle aspiration, Milan System, ROSE, telecytology, cell block, core needle biopsy, immunohistochemistry, FISH, and next-generation sequencing. Priority was given to recent meta-analyses, multicenter cohorts, national or large institutional validation studies, and papers that explicitly reported ROM and follow-up methodology. Smaller or single-institution series were retained only when they addressed specific practice problems that are underrepresented in large datasets—such as lymphoid-rich aspirates, cystic lesions, or metastases to the parotid—and their conclusions are explicitly framed as context-limited rather than definitive.

### Milan System 2nd edition

MSRSGC was designed to address a pragmatic clinical need: salivary gland cytology required a universal reporting structure that could communicate uncertainty, attach evidence-based ROM values, and inform clinical management. The MSRSGC emerged from an international effort led by the American Society of Cytopathology and the International Academy of Cytology to standardize terminology and link cytology categories to ROM and management guidance. The six diagnostic categories (non-diagnostic; non-neoplastic; atypia of undetermined significance (AUS); neoplasm (further split into benign and salivary gland neoplasm of uncertain malignant potential (SUMP)); suspicious for malignancy; malignant) provide that structure and have been validated across multiple cohorts [6, 8, 9].

Building on the first edition, the Milan System 2nd edition preserves the original framework and reporting format, while introducing evidence-based refinements and practical updates on imaging correlation and ancillary testing. It refines ROM values using broader evidence, integrates a dedicated imaging perspective, and expands practical guidance on ancillary testing, reflecting how clinical decision-making increasingly depends on subtyping, grading, and, in selected cases, predictive biomarkers. The practical implication is that categories are not endpoints. Indeterminate categories (particularly AUS and SUMP) should trigger deliberate workflow responses (improved targeting, ancillary triage, or tissue escalation) rather than passive acceptance of uncertainty.

Milan System 2nd edition also reinforces several “high-impact” ROM messages that are easy to miss in day-to-day practice. In particular, the ROM associated with a “non-diagnostic” (ND) result remains non-trivial (approximately one quarter in many series), underscoring that insufficient material should not be misconstrued as reassuring, especially in cystic or heterogeneous lesions where low-grade malignancies may yield paucicellular fluid. Conversely, a definitive “malignant” cytologic diagnosis carries an extremely high ROM (often > 98%), supporting confident escalation of surgical planning when the sample is representative [6].

**Fig. 1:**
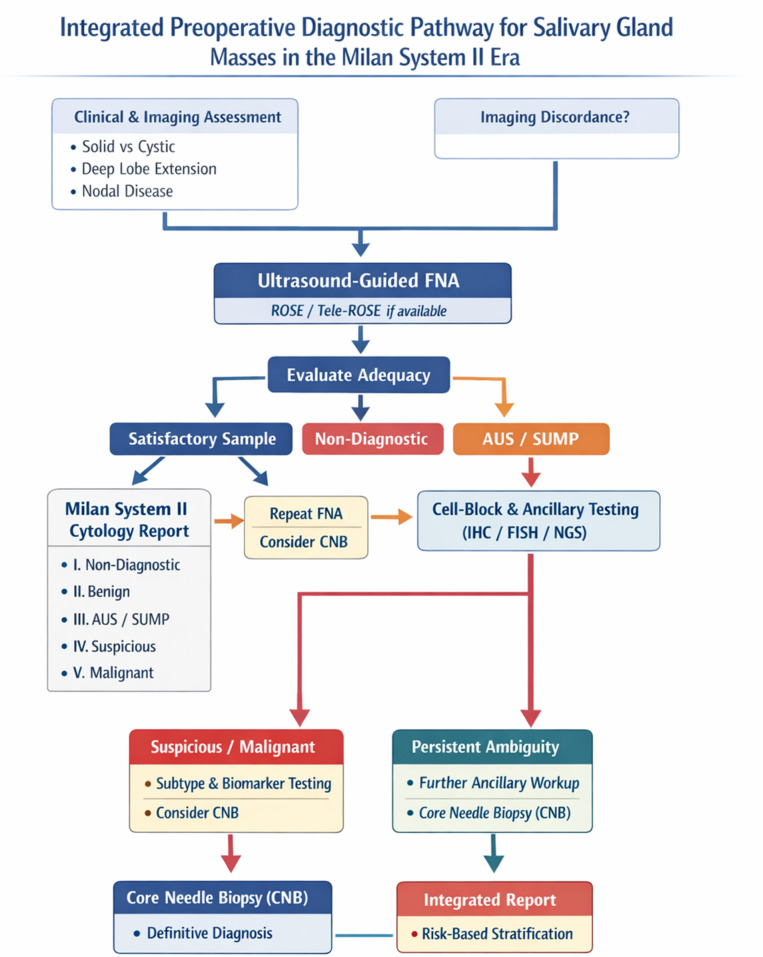
Integrated preoperative diagnostic pathway for salivary gland nodules: a Milan System 2nd edition–based approach. Clinical context and imaging are integrated upfront to guide targeting and escalation. First-line sampling is ultrasound-guided fine-needle aspiration (FNA), with rapid on-site evaluation (ROSE) or tele-rapid on-site evaluation (tele-ROSE), when available, to assess adequacy and triage material for cell-block and ancillary testing. Results are reported using the Milan System 2nd edition as a risk- and management-oriented message; a non-contributory cell-block is documented as a clinically relevant limitation. Escalation is driven by Milan category, level of suspicion, and clinical impact: non-diagnostic results prompt repeat targeted FNA, with preference for core needle biopsy (CNB) when inadequacy persists or suspicion remains high; atypia of undetermined significance (AUS) or salivary gland neoplasm of uncertain malignant potential (SUMP) triggers problem-solving on cell-block with immunohistochemistry (IHC), fluorescence in situ hybridization (FISH), and/or next-generation sequencing (NGS), with CNB favored when ambiguity persists and histotype or grade would affect surgical planning. Suspicious/malignant categories prompt ancillary refinement (subtyping, grading, predictive biomarkers) and CNB when cytology cannot support the required work-up or when architecture is needed; if CNB is non-specific, Milan-like risk stratification can standardize communication within the integrated pathway

In parallel, Milan System 2nd edition shifts adequacy from a rigid numeric threshold toward a qualitative, context-dependent concept: a specimen is “adequate” if it provides sufficient material for an informative interpretation in the given clinical–radiologic scenario. Practically, this means that an apparently “low-cellularity” aspirate may still be adequate for a specific management question in selected contexts, whereas a cyst-fluid–only aspirate in a solid mass should lower the threshold for repeat sampling or escalation (Fig. 1).

### Interpreting ROM in practice: benchmark values, denominator effects, and interinstitutional variability

The benchmark ROMs adopted in the Milan System 2nd edition should be interpreted as evidence-based category anchors rather than as immutable values applicable to every setting. The second edition explicitly refined ROM estimates on the basis of broader validation data, including systematic reviews and meta-analyses, while preserving the original management-oriented logic of the system [6, 8]. In this context, the most comprehensive pooled analysis is a meta-analysis by Wang et al., which included 35 studies, 10,706 salivary gland FNAs, and 7168 cases with histopathologic follow-up; the pooled ROMs were 11.4% for nondiagnostic, 10.9% for non-neoplastic, 30.5% for AUS, 2.8% for benign neoplasm, 37.7% for SUMP, 83.8% for suspicious for malignancy, and 97.7% for malignant [10]. Importantly, low-level heterogeneity was observed overall, supporting the general robustness of the Milan framework while still leaving room for category-specific variability across practice settings. Jalaly et al. similarly concluded, in a broad literature review, that the Milan system performs well as a standardized reporting framework, but that institutional case mix, follow-up structure, and local diagnostic thresholds remain important when individual ROM values are interpreted in daily practice [11].

A major reason ROM may vary across reports is the denominator used for its calculation. ROM derived only from resected or otherwise tissue-confirmed cases is vulnerable to enrichment for clinically worrisome lesions, whereas ROM based on the entire cytologic category, including non-operated cases with clinical follow-up, may better reflect day-to-day practice. This issue is particularly well illustrated for the nondiagnostic category. In a literature review of 50 Milan studies, Lui et al. showed that for nondiagnostic salivary gland FNAs the ROM based on cases with surgical/flow-cytometric follow-up (FROM) was 15.7%, whereas the overall ROM (OROM) across the entire category was only 4.1%; both values were lower than the corresponding “all-comer” malignancy rates [12]. A similar denominator effect is evident in AUS. In the bi-institutional series by Alruwaii et al., the estimated ROM for AUS was 37.5% when only cases with tissue follow-up were considered, but 25.0% when the entire AUS cohort was used as denominator [13]. Accordingly, when benchmark ROMs are discussed, it is useful to specify whether the quoted value is based on resection-only follow-up or on a broader category denominator, because the two are not interchangeable and may communicate different clinical messages.

A second practical modifier of ROM is interinstitutional variability, which is not uniform across all Milan categories. Some categories are relatively stable across centers, whereas others are more sensitive to interpretive thresholds, case mix, and local expertise. For example, Wang et al. showed marked interinstitutional variability in the “atypical”/AUS-type category, with ROM among five tertiary centers ranging from 73.08% to 0.00%, suggesting that this category is especially vulnerable to institutional differences in diagnostic practice [14]. By contrast, Maleki et al. found that the suspicious-for-malignancy category was much more homogeneous: the ROM was 83.3% overall and ranged from 74 to 88% across five institutions, without significant interinstitutional differences [15]. Additional evidence indicates that variability is also influenced by organization and technical workflow. Reerds et al. showed in Dutch clinical practice that the sensitivity of parotid FNAC was highest in dedicated head-and-neck oncology centers (88.1%), lower in affiliated centers (79.7%), and lowest in general hospitals (75.0%), underscoring the contribution of referral pattern and subspecialty expertise [16]. Higuchi et al., in a 12-institution Japanese study, similarly confirmed that the Milan system remains valid in multicenter practice while also showing that institutions using Romanowsky-stained preparations had lower nondiagnostic rates and lower ROM in the non-neoplastic category [17].

Taken together, these data support a practical reading of ROM in the Milan System 2nd edition: benchmark values should guide communication and management, but they should be interpreted alongside three modifiers: the denominator used, the category involved, and the institutional setting in which the result was generated. This is particularly relevant for nondiagnostic and AUS/SUMP interpretations, in which re-biopsy decisions, ancillary triage, and escalation to core needle biopsy may depend not only on the nominal category ROM, but also on whether the reported risk derives from resection-enriched cohorts and whether the local institution has demonstrated stable category performance. In contrast, the suspicious-for-malignancy and malignant categories remain comparatively robust across series and therefore carry more reproducible preoperative risk messaging. In addition to pooled evidence and multicenter validation studies, category-focused institutional series remain useful for practical interpretation of indeterminate Milan categories; in particular, Cormier and Agarwal provide focused evidence on the morphologic and histologic correlates that underlie AUS and SUMP assignments in routine practice, thereby complementing benchmark ROM estimates and denominator-based analyses [18]. In parallel, implementation-oriented cohorts also add practical context to category performance; notably, Morand et al. demonstrated the feasibility of applying MSRSGC principles on cell-block material, thereby reinforcing the practical relevance of cell-block-based classification and ancillary testing in routine salivary gland cytopathology [19]. Representative single-institution validation studies can also add practical context to category performance across the Milan spectrum; notably, Park et al. reported an overall diagnostic accuracy of 95.9% and a ROM of 2.5% for the benign neoplasm category, supporting the reproducibility of low-risk performance in category IVa in routine practice [9]. Table 1 summarizes Milan System 2nd edition benchmark ROM values together with the principal benchmark sources, pooled supporting evidence, and representative cohort studies illustrating denominator-dependent, interinstitutional, implementation, and category-specific contextual variation.

**Table 1:** Practical interpretation of Milan System 2nd edition ROM: benchmark values, supporting evidence, and representative cohort effects

MSRSGC category	Milan System 2nd edition benchmark ROM	Principal benchmark source	Pooled/supporting evidence	Representative cohort(s) illustrating denominator, interinstitutional, implementation, or category-specific contextual effects	Practical note for interpretation
I. Nondiagnostic (ND)	15%	*The Milan System for Reporting Salivary Gland Cytopathology*, 2nd ed. [6]; Rossi et al. 2024 [8]	Wang et al. 2022: pooled ROM 11.4% [10]; Jalaly et al. 2020: mean ROMs in most studies fall within Milan ranges [11]	Lui et al. 2022: FROM 15.7% vs OROM 4.1% [12]; Higuchi et al. 2022: multicenter application showed lower ND rates in institutions using Romanowsky-stained preparations [17]	ROM is strongly denominator-sensitive; resection-only follow-up may overestimate day-to-day risk
II. Non-neoplastic	11%	*The Milan System for Reporting Salivary Gland Cytopathology*, 2nd ed. [6]; Rossi et al. 2024 [8]	Wang et al. 2022: pooled ROM 10.9% [10]; Jalaly et al. 2020: mean ROMs in most studies fall within Milan ranges [11]	Reerds et al. 2021: ROM 10.3% in routine practice; Higuchi et al. 2022: lower ROM in this category in institutions using Romanowsky-stained preparations [17]	A low ROM should not obscure false-negative cystic or low-grade malignancies; local workflow influences performance
III. AUS	30%	*The Milan System for Reporting Salivary Gland Cytopathology*, 2nd ed. [6]; Rossi et al. 2024 [8]	Wang et al. 2022: pooled ROM 30.5% [10]; Jalaly et al. 2020: mean ROMs in most studies fall within Milan ranges [11]	Alruwaii et al. 2020: estimated ROM 37.5% vs overall ROM 25.0% [13]; Wang et al. 2017: interinstitutional ROM range 73.08% to 0.00% [14]; Cormier and Agarwal 2022: category-focused institutional series highlighting the morphologic and histologic correlates that drive AUS assignment in routine practice [18]	AUS is one of the most context-dependent categories; denominator effects, institutional thresholds, and morphologic context should all be acknowledged explicitly
IVa. Benign neoplasm	< 3%	*The Milan System for Reporting Salivary Gland Cytopathology*, 2nd ed. [6]; Rossi et al. 2024 [8]	Wang et al. 2022: pooled ROM 2.8% [10]; Jalaly et al. 2020: mean ROMs in most studies fall within Milan ranges [11]	Reerds et al. 2021: ROM 2.3% [16]; Park et al. 2020: single-institution validation; overall accuracy 95.9%, ROM 2.5% for benign neoplasm [9]	This is one of the most stable categories; ROM is generally reproducible across series
IVb. SUMP	35%	*The Milan System for Reporting Salivary Gland Cytopathology*, 2nd ed. [6]; Rossi et al. 2024 [8]	Wang et al. 2022: pooled ROM 37.7% [10]; Jalaly et al. 2020: mean ROMs in most studies fall within Milan ranges [11]	Reerds et al. 2021: ROM 28.6% [16]; Morand et al. 2022: cell-block-based MSRSGC implementation cohort with ROM 30% for SUMP [19]; Cormier and Agarwal 2022: institutional evidence emphasizing the histologic correlates and practical diagnostic contexts of SUMP in routine practice [18]	SUMP remains a heterogeneous category in which architecture, ancillary studies, local expertise, implementation setting, and category-defining morphologic context materially influence downstream ROM
V. Suspicious for malignancy	83%	*The Milan System for Reporting Salivary Gland Cytopathology*, 2nd ed. [6]; Rossi et al. 2024 [8]	Wang et al. 2022: pooled ROM 83.8% [10]; Jalaly et al. 2020: mean ROMs in most studies fall within Milan ranges [11]	Maleki et al. 2018: overall ROM 83.3%, with no significant interinstitutional variability [15]; Reerds et al. 2021: ROM 83.0% [16]	This is a comparatively robust category with reproducible risk messaging across institutions
VI. Malignant	> 98%	*The Milan System for Reporting Salivary Gland Cytopathology*, 2nd ed. [6]; Rossi et al. 2024 [8]	Wang et al. 2022: pooled ROM 97.7% [10]; Jalaly et al. 2020: mean ROMs in most studies fall within Milan ranges [11]	Reerds et al. 2021: ROM 99.3% [16]; Morand et al. 2022: cell-block-based implementation cohort with ROM 100% in the malignant category [19]	This is the most stable category; variability is limited and the main issue becomes subtype refinement rather than basic ROM communication

*MSRSGC* Milan System for Reporting Salivary Gland Cytopathology, *ROM* risk of malignancy, *ND* nondiagnostic, *AUS* atypia of undetermined significance, *SUMP* salivary gland neoplasm of uncertain malignant potential, *FROM* risk of malignancy calculated on cases with tissue or flow-cytometric follow-up, *OROM* overall risk of malignancy calculated on the entire cytologic category

### Sample optimization

In salivary gland cytology, the boundary between diagnostic and non-diagnostic is frequently determined before a slide reaches the microscope. Sampling inadequacy drives repeat procedures and diagnostic delay, especially in cystic lesions, necrotic tumors, and heterogeneous nodules [4]. ROSE improves this vulnerability by allowing real-time assessment of adequacy and immediate adjustment of technique and targeting, thereby reducing non-diagnostic outcomes and enabling purposeful triage of additional passes toward cell-block or other ancillary workflows [2, 20].

Evidence supporting ROSE in salivary FNA includes studies demonstrating meaningful agreement between ROSE impressions and final cytologic diagnoses, supporting ROSE as a practical tool to optimize sampling quality [2]. The extension of ROSE through telecytology offers a scalable solution where on-site cytopathology staffing is limited. Tele-ROSE studies emphasize the importance of quality assurance, particularly auditing cases deemed adequate on-site but ultimately non-diagnostic on final evaluation, to refine triage protocols and training [20, 21]. Within a Milan System 2nd edition pathway, ROSE/tele-ROSE strengthens the reliability of category assignment while increasing the likelihood that a single minimally invasive procedure yields an “actionable” outcome [2, 6, 8, 20].

## Ancillary readiness: cell-block

Cell-block preparation converts residual aspirated material into Formalin-Fixed Paraffin-Embedded (FFPE) tissue, enabling histology-like sections, standardized IHC, FISH, and compatibility with many molecular workflows. In salivary gland pathology, where lineage assignment, subtyping, and grade often depend on immunophenotypic and molecular features, cell-block transforms FNA into an “ancillary-ready” test rather than a stand-alone smear-based assessment. Indeed, the transition to this ancillary-ready paradigm heavily relies on specimen adequacy; recent international multi-institutional evidence highlights that the cellularity of routinely prepared cell-blocks is a critical determinant for the success of downstream testing, shifting the focus from simple preparation to rigorous quality assessment [22].

Multiple cohorts show that cell-block contributes meaningfully to diagnosis and improves the practical performance of MSRSGC reporting [19, 23]. A key interpretive insight emerges from Tommola and colleagues: non-contributory cell-blocks were associated with higher false-negative/indeterminate outcomes, implying that an uninformative cell-block is frequently a proxy for sampling limitations rather than a neutral technical event [23]. In a Milan System 2nd edition-driven workflow, this finding is actionable: a non-contributory cell-block should prompt transparent reporting of the limitation and consideration of escalation (repeat targeted FNA with ROSE/tele-ROSE or CNB when clinical suspicion/management impact is high) [6, 23].

Morand and colleagues further demonstrated the feasibility of applying MSRSGC principles using cell-block FFPE material, reinforcing the concept that cell-block can serve as a robust substrate for standardized classification and ancillary testing when implemented systematically [19].

### Focused ancillary testing

Milan System 2nd edition recognizes that salivary cytology increasingly intersects with precision-oriented questions. While many cases can be managed based on ROM and classic morphologic diagnosis, selected scenarios require additional information: confirmation of lineage, resolution of primary versus metastasis, refinement of low- versus high-grade behavior, and, in advanced disease, identification of potentially actionable alterations [6]. Ancillary testing is frequently informative when applied strategically. Consistent with this escalation logic, ASCO guidelines recommend preoperative tissue biopsy (either fine-needle aspiration biopsy (FNAB) or CNB) to differentiate salivary gland malignancies from non-malignant lesions, and explicitly support CNB when FNAB proves inadequate or when the subsite precludes FNAB (e.g., deep minor salivary glands) [3]. The same guidance highlights the role of ancillary testing (IHC and/or molecular studies) on both FNAB and CNB material to support diagnosis and refine risk stratification (ROM) when clinically relevant [3]. In a four-year MSRSGC-based cohort, Dubucs and colleagues showed that immunocytochemistry and FISH contributed to a definitive diagnosis in a meaningful subset of selected cases, particularly when tumor features suggested a resolvable differential diagnosis [24]. However, these benefits depend on material sufficiency and appropriate processing: exactly what ROSE-guided triage and cell-block workflows are designed to secure [2, 20, 24].

A clinically grounded cell-block strategy relies on focused panels rather than indiscriminate testing. In this setting, evidence that supports specific markers for specific differential diagnoses is particularly valuable.

From a practical standpoint, ancillary testing resolves a substantial fraction of AUS/SUMP by converting pattern-based differentials into lineage-anchored or fusion-anchored diagnoses. A small number of high-yield immunomarkers can be particularly impactful in common “gray-zone” scenarios: PLAG1 and HMGA2 support pleomorphic adenoma and carcinoma ex pleomorphic adenoma in basaloid neoplasms; MYB nuclear staining serves as a useful surrogate for *MYB*-rearranged adenoid cystic carcinoma in difficult cases; DOG1 can serve as a useful adjunct in diagnosing acinic cell carcinoma when acinic differentiation is in the differential; and androgen receptor (AR) with HER2 testing helps confirm salivary duct carcinoma while simultaneously informing potential targeted therapy options [25–29].

Likewise, recurrent gene fusions increasingly define diagnostically challenging entities and can be interrogated on cell-block/CNB when morphology is limited: ETV6–NTRK3 supports secretory carcinoma (with distinct therapeutic implications in the TRK inhibitor era); CRTC1–MAML2 supports mucoepidermoid carcinoma and may be particularly useful in cystic, paucicellular specimens; EWSR1–ATF1 supports hyalinizing clear cell carcinoma; and MYB-NFIB/MYBL1-NFIB support adenoid cystic carcinoma [28, 30–32].

FISH remains particularly useful for signature rearrangements in selected salivary tumors, and molecular workflows are expanding as laboratories adopt targeted strategies feasible on cell-block or small biopsy FFPE material [24, 33]. Recent feasibility work supports the practical detection of relevant fusions and alterations from limited specimens using RNA-based NGS approaches, providing a path toward integrating molecular signatures into diagnostic classification and, where appropriate, treatment selection [33]. In parallel, experience from other cytology-based standardized systems shows that focused (“limited but well-chosen”) molecular testing can meaningfully improve diagnostic/prognostic stratification when aligned with workflow triage and clinical need [34]. Furthermore, the reliability of cytologic and cell-block material extends to the evaluation of predictive biomarkers. Recent large-scale international studies have demonstrated high interobserver agreement in biomarker scoring (such as PD-L1) on cytology, solidifying the role of FNA as a robust standalone tool for precision oncology planning [35].

### FNA versus CNB

Even with best-practice cytology workflows, some questions exceed what limited cytologic sampling can answer. Architecture, stromal context, and broader tissue representation may be required to confidently subtype or grade malignancies, characterize lymphoid processes, or provide enough material for extensive ancillary testing [36–39]. CNB addresses these gaps by providing intact tissue cores under ultrasound guidance.

Meta-analyses have reported high diagnostic performance for ultrasound-guided CNB, with sensitivities in the low-to-mid 90% range and specificities approaching 99–100% in many series; careful knowledge of intraparotid vascular anatomy and real-time ultrasound guidance are key to minimizing hematoma risk and avoiding nerve-adjacent trajectories [4, 37–39].

Recent single-institution cohorts directly comparing modalities provide concrete benchmarks for the added value of CNB in salivary gland lesions. In a 5-year North Indian experience, Chowdhury et al. reported concordance between FNAC and CNB diagnoses in 78.1% of cases, whereas CNB showed 100% concordance with the final diagnosis on subsequent resection [5]. In a parotid-gland CNB series, Israel and Griffith found that CNB yielded a specific diagnosis in 63% of cases, with 94% accuracy among those cases with surgical follow-up [40]. Taken together, these data support CNB as a selective escalation step when cytology is limited by sampling or when architectural assessment and more definitive preoperative subtyping are expected to influence operative planning and ancillary work-up.

Comparative studies consistently indicate that CNB improves sensitivity and subtyping, particularly for malignant tumors, over FNA, while maintaining a favorable safety profile [36, 38]. Systematic reviews and meta-analyses have long supported the high diagnostic accuracy of ultrasound-guided CNB for salivary gland lesions [38, 39]. More recently, a dedicated meta-analysis comparing FNA and CNB in salivary gland tumors reported superior diagnostic performance and procedural outcomes for CNB, including lower non-diagnostic rates and fewer repeat procedures, with complications generally uncommon and most often limited to minor hematoma [36]. These findings support a selective CNB strategy: CNB should not replace FNA as universal first-line testing, but should be considered early when repeated non-diagnostic results occur, when AUS/SUMP persists with major management implications, or when architectural evaluation and tissue volume are clinically pivotal [36, 38]. Beyond diagnostic performance, CNB can improve pathway efficiency. Lower inadequacy rates translate into fewer repeat procedures and shorter time-to-decision. In addition, when CNB delivers a definitive malignant diagnosis preoperatively, it may reduce the likelihood of ‘two-stage’ surgical scenarios (e.g., an initial diagnostic parotidectomy followed by completion surgery once malignancy is identified), with potential benefits in cost containment and patient morbidity [37].

A practical barrier to CNB adoption is reporting heterogeneity. Applying a Milan-like risk stratification concept to parotid CNB has been shown to be feasible and clinically informative, supporting standardized communication when CNB yields limited tissue or when a categorical risk statement better serves multidisciplinary planning [40, 41]. Such harmonization is especially valuable for integrated pathways that combine FNA, CNB, and resection data to track ROM/RON (risk of neoplasm) outcomes across modalities [40, 41].

Two historical concerns have limited broader uptake of parotid CNB (i.e., facial nerve injury and needle-tract seeding), but modern ultrasound-guided techniques have largely reframed both as low-probability events. Systematic reviews across large cohorts report no permanent facial nerve paralysis attributable to ultrasound-guided CNB, plausibly reflecting real-time visualization that allows operators to avoid major vessels and nerve-adjacent trajectories [37, 42]. Similarly, the risk of tumor seeding appears negligible in contemporary series; critically, the theoretical risk is outweighed by the clinical benefit of a definitive preoperative diagnosis when it enables single-stage, properly planned surgery rather than staged escalation after an indeterminate work-up [43].

For practical use in routine preoperative salivary gland diagnostics, Table 2 summarizes common high-impact preoperative scenarios in salivary gland cytology and aligns each with the most useful next procedural step, the preferred ancillary or tissue-escalation strategy, and the main practical rationale. The emphasis is not on category labeling alone, but on how nondiagnostic, indeterminate, lymphoid-rich, cystic, metastatic, and overtly malignant scenarios can be managed within an integrated pathway combining ultrasound-guided FNA, ROSE/tele-ROSE, cell-block preparation, ancillary testing, and selective escalation to CNB.

**Table 2:** Practical escalation matrix for preoperative salivary gland diagnostics in a Milan System 2nd edition–based workflow

Clinical/cytologic scenario	Main practical pitfall	Most useful immediate next step	Preferred ancillary/escalation strategy	Practical rationale
ND aspirate (including cyst-fluid only or markedly paucicellular samples)	False reassurance from insufficient material; repeat sampling may reproduce the same inadequacy if targeting is unchanged	Repeat targeted ultrasound-guided FNA, ideally with ROSE/tele-ROSE	Prepare cell-block if any material is available; proceed to CNB if inadequacy persists or clinicoradiologic suspicion remains high	A nondiagnostic result should be treated as a sampling failure rather than as a low-risk outcome; real-time adequacy assessment and improved targeting are the most effective first corrections
AUS with limited atypia and/or suboptimal representativeness	Indeterminate interpretation driven by threshold issues, scant lesional cells, blood/cystic dilution, or poorly contributory cell-block	Improve adequacy and representativeness at the next sampling event	Repeat FNA with ROSE/tele-ROSE plus ancillary-ready cell-block; consider CNB when management would change on the basis of a more definitive classification	In AUS, the highest-yield intervention is often better sampling and better triage of material, rather than immediate repetition of the same low-information pathway
SUMP/clearly neoplastic sample lacking reliable benign–malignant adjudication	Cytology establishes neoplasia but cannot resolve architecture, invasion-related implications, or decisive malignant features	Prioritize clarification rather than simple repetition	Cell-block-based IHC/FISH/NGS when adequate; early CNB when architecture, histotype refinement, or grading would influence treatment planning	In SUMP, repeated aspirates may reproduce the same ambiguity; escalation should be driven by the need for architecture and management-relevant subclassification
Cystic salivary lesion with macrophage-rich/proteinaceous aspirate	Under-sampling of the lesional epithelial component; benign-appearing cyst contents may mask neoplasm or malignancy	Re-target the solid mural component/thickened wall during the same or repeat procedure	ROSE/tele-ROSE to identify “cyst contents only”; cell-block for residual epithelial fragments; CNB if the solid component remains difficult to sample or suspicion stays high	Cystic change is a major driver of false-negative and nondiagnostic results; active image-guided targeting is more important than simply repeating aspiration
Lymphoid-rich aspirate	Reactive lymphoid tissue, autoimmune-related lymphoid proliferation, and lymphoma may overlap cytologically; delayed triage can waste diagnostic material	Treat the case as a triage event at first encounter	Preserve material for flow cytometry, cell-block, and, where appropriate, molecular clonality testing; use CNB when architecture is required for classification	The key step is front-loaded triage: if atypical lymphoid material is recognized early, the pathway can shift from descriptive cytology to definitive hematopathology work-up
Possible metastasis versus primary salivary gland lesion	Cytomorphologic overlap may lead to misclassification; failure to integrate clinical history can delay the correct work-up	Correlate immediately with clinical history and imaging	Cell-block with targeted IHC first; CNB when phenotype remains unresolved or more tissue is needed for lineage confirmation	The practical question is often not merely “malignant or not,” but “primary salivary versus metastatic,” which requires phenotype-oriented triage and adequate FFPE material
Suspicious for malignancy/malignant cytology requiring subtype, grade, or predictive refinement	Cytology may establish malignancy but still be insufficient for grading, full subtyping, or biomarker testing relevant to surgery or oncology planning	Convert a “malignant” result into a management-ready result	Use cell-block for IHC/FISH/NGS when feasible; add CNB when architecture, grading, or broader biomarker work-up is needed	In high-risk categories, the goal is not simply confirmation of malignancy, but production of the level of diagnostic detail needed for treatment planning

*FNA* fine-needle aspiration, *ROSE* rapid on-site evaluation, *tele-ROSE* tele-rapid on-site evaluation, *CNB* core needle biopsy, *AUS* atypia of undetermined significance, *SUMP* salivary gland neoplasm of uncertain malignant potential, *IHC* immunohistochemistry, *FISH* fluorescence in situ hybridization, *NGS* next-generation sequencing, *FFPE* formalin-fixed paraffin-embedded

## Discussion 

Milan System 2nd edition encourages the field to evaluate preoperative salivary diagnostics not only by slide-level accuracy but by pathway performance: how often the initial procedure yields a clinically actionable result, how often repeat sampling is required, and how uncertainty is managed when it matters most [6, 8]. From this perspective, the most impactful improvements are practical and scalable. ROSE/tele-ROSE reduces sampling noise and enables intelligent triage [2, 20, 21]. Cell-block transforms cytology into an FFPE platform capable of standardized IHC/FISH/NGS, and evidence indicates that a non-contributory cell-block should itself influence management because it signals a higher risk of false reassurance [19, 23]. CNB complements this strategy by providing architecture and tissue volume when needed, with consistent evidence for superior sensitivity and adequacy compared with FNA and with low rates of significant complications [36, 38, 39].

Ancillary testing is the bridge between Milan categories and modern clinical needs. Immunophenotypic and molecular tools can resolve high-impact differentials (primary versus metastasis, epithelial versus lymphoid, low versus high grade) when the substrate is sufficient and the testing is question-driven [24, 33]. The emerging feasibility of RNA-based NGS approaches on small specimens further expands the ability to deliver classification and, in selected cases, actionable information from minimal sampling [33]. Nonetheless, the guiding principle remains disciplined triage: focused ancillary testing is most effective when performed because it changes management, not simply because it is available.

### AUS versus SUMP

AUS and SUMP are often discussed as if they were simply “indeterminate” bins, but in practice they represent different kinds of uncertainty that arise from distinct failure modes. AUS is frequently a sampling- and threshold-driven category: the aspirate may be scant, obscured by blood or cyst contents, or show limited atypia that cannot be confidently attributed to a neoplasm. SUMP, in contrast, typically reflects a sample that is clearly neoplastic yet resists benign–malignant adjudication because cytomorphology alone cannot reliably capture invasion, architecture, or decisive high-grade features. This distinction matters because the most effective pathway response differs. In AUS, the highest-yield intervention is often improving adequacy and representativeness (better targeting, more passes, ROSE/tele-ROSE), and ensuring an ancillary-ready cell-block so that the same sampling event can be “upgraded” if needed. In SUMP, the highest-yield step is often ancillary clarification (IHC/FISH/NGS on cell-block) or, when architecture/subtyping is critical for management, early escalation to CNB, rather than repeated aspirates that risk reproducing the same interpretive ambiguity. The performance gap between “determinate” and “indeterminate” categories is consistently recognized in the evidence synthesis you provided (with indeterminate categories showing lower accuracy than determinate ones), reinforcing that AUS/SUMP are not merely semantic labels but markers of where the pathway must adapt [8, 18, 44, 45].

Additional practical insight into these indeterminate categories is provided by the institutional study of Cormier and Agarwal, which specifically examined the incidence and histologic correlates of AUS and SUMP and showed that the value of these categories lies not only in ROM stratification, but also in clarifying the morphologic contexts in which indeterminate interpretations arise in routine practice [18].

A practical and underused concept is that the quality of the cell-block itself can help triage AUS/SUMP. When the cell-block is non-contributory (insufficient FFPE material), studies show a higher likelihood of false-negative or persistently indeterminate outcomes, effectively making a failed cell-block a “sampling alarm.” In Milan System 2nd edition-driven reporting, explicitly noting a non-contributory cell-block is not just transparency: it is a pathway trigger that supports either targeted repeat FNA (ideally ROSE-guided) or CNB depending on clinical–radiologic suspicion and the management stakes [23]. In the same practical direction, Morand et al. showed that MSRSGC principles can be successfully applied on cell-block material, supporting the view that cell-block is not merely ancillary support, but can also serve as a practical substrate for standardized classification and management-oriented reporting in selected salivary gland cases [19].

### Cystic lesions

**Fig. 2:**
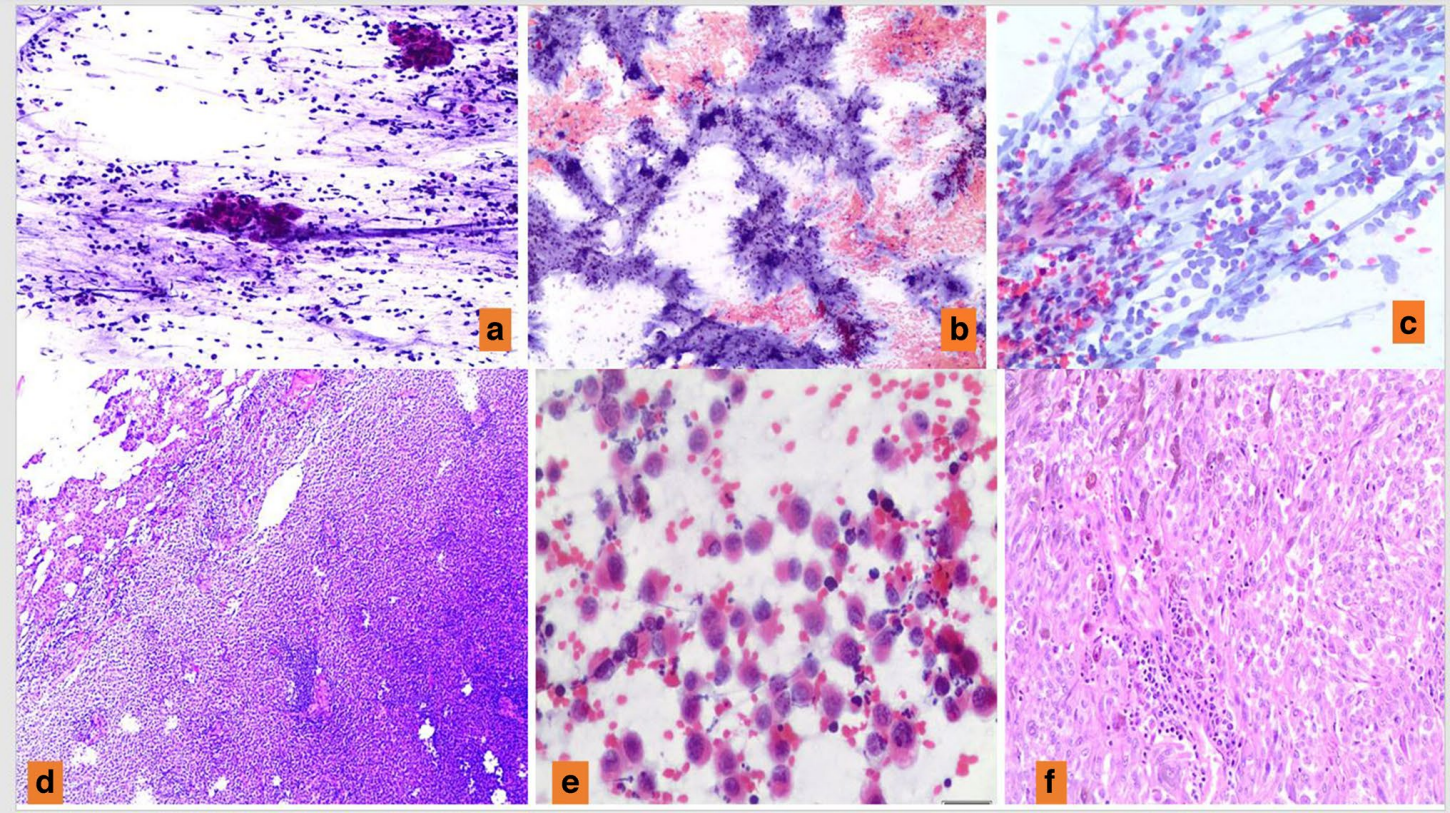
Composite figure illustrating representative diagnostic situations in preoperative salivary gland cytology. **a** A cystic/oncocytic lesion represented by Warthin tumor, characterized by a dirty background, lymphocytes, and oncocytic cells (Pap stain, ×40). **b** A classic pleomorphic adenoma pattern, with chondro-myxoid stromal matrix, myoepithelial cells with variable morphology, and small clusters of ductal cells (Pap stain, 40x). **c** and **d** A lymphoid-rich salivary gland lesion: **c** The cytologic features of a parotid non-Hodgkin lymphoma, with a diffuse proliferation of small- to medium-sized lymphocytes and atypical nuclear features (Pap stain, ×40). **d** The corresponding histologic biopsy confirming non-Hodgkin B-cell lymphoma (H&E, ×40). **e** and **f** Metastatic involvement of the parotid gland by melanoma, showing discohesive or loosely cohesive atypical cells with prominent nucleoli and melanin pigment, both intracellularly and in the background (Pap stain, ×40)

Cystic change is one of the most important practical confounders in salivary gland sampling because it can dilute lesional cells, inflate the non-diagnostic rate, and generate deceptively bland cytology. A familiar pitfall is the cystic/oncocytic spectrum: aspirates dominated by macrophages, proteinaceous fluid, and scant oncocytic epithelium can be reported as non-diagnostic or AUS, despite the underlying lesion being a Warthin tumor or another oncocytic neoplasm, as illustrated in Fig. 2a. By contrast, a representative benign neoplasm pattern with abundant diagnostic matrix and lesional cells, such as pleomorphic adenoma, is illustrated in Fig. 2b and highlights how sample representativeness directly affects interpretive confidence and downstream triage [2, 46, 47].

The more consequential risk, however, is the cystic presentation of malignancy, particularly when only cyst fluid is sampled. In this setting, repeated “benign” or “non-diagnostic” FNAs can occur unless imaging is actively used to target the solid mural component or thickened wall. This is exactly where ROSE/tele-ROSE and imaging-informed targeting become pathway-critical: if the on-site impression is essentially “cyst contents only,” the procedure should be extended (additional passes, altered target) during the same session rather than deferring to a second visit. When the solid component remains difficult to sample or the clinical suspicion is high despite repeated non-representative aspirates, CNB becomes a rational escalation because it is more likely to capture the diagnostic architecture and the cellular compartment of interest [36, 40, 48].

### Lymphoid-rich aspirates

Lymphoid-rich salivary aspirates sit at a crossroads of benign inflammatory processes, autoimmune disease-related lymphoid proliferations, and true lymphomas. The cytologic overlap can be substantial, and the consequences of mis-triage are real: a lymphoma may be underestimated on cytology if interpreted as reactive lymphoid tissue, whereas correlation with tissue findings may confirm a true lymphoproliferative disorder, as illustrated in Fig. 2c and d. The most “practice-changing” element is not a single cytologic criterion, but front-loaded triage: when ROSE/tele-ROSE identifies a lymphoid-dominant sample with atypical features, the sampling strategy should be immediately adapted to preserve material for ancillary hematopathology work-up. The evidence synthesis you shared highlights that, in suspected salivary gland lymphoma, molecular clonality testing on cytologic material (e.g., immunoglobulin gene rearrangement PCR) can support diagnosis without waiting for excision, but this is only possible when the pathway anticipates the need and preserves adequate material [49, 50].

CNB is particularly valuable in this setting when architecture is needed for classification or when cytology provides suspicion but not sufficient resolution. The key is to avoid a “serial FNA loop” that keeps generating limited lymphoid material without enabling definitive subclassification. A Milan System 2nd edition-driven approach is to treat a lymphoid-rich aspirate as a triage event: either secure the ancillary substrate at the first encounter (cell-block + molecular/flow pathways where available) or escalate early to CNB when the downstream decision depends on tissue architecture [36, 40]. Available salivary-specific evidence in this setting is still derived largely from retrospective single-center or otherwise limited cohorts, so these data are best used to inform triage rather than as stand-alone quantitative benchmarks [49, 50].

### Metastatic work-up

Metastatic disease to the salivary glands (particularly to the parotid) creates a recurring diagnostic trap because the gland can be both a primary site and a recipient of metastases, and because some metastases can mimic primary salivary malignancies cytologically, as illustrated in Fig. 2e and f. The two classic pathway errors are (1) interpreting a metastasis as a primary salivary carcinoma (leading to incomplete staging or misdirected therapy), and (2) assuming a lesion is metastatic without adequate confirmation (leading to inappropriate management). The practical solution again is pathway-based rather than purely morphologic: the diagnostic team should explicitly keep metastasis in the differential when clinical history, imaging distribution, or cytologic phenotype suggests it, and should use cell-block-enabled IHC strategically to establish lineage and site-consistent immunoprofiles. Your evidence synthesis explicitly notes that ancillary testing on cytology is particularly useful to avoid errors such as mistaking metastasis for a salivary primary, and it frames IHC/FISH as tools to “de-risk” these clinically high-impact scenarios [51, 52].

When cytology remains equivocal, either because the phenotype is ambiguous, the cell-block is non-contributory, or the needed marker/molecular work-up exceeds the available material, CNB offers a practical escalation because it increases tissue volume and supports broader, more reproducible ancillary testing. Importantly, this escalation should be guided by the management question: if confirming metastatic disease will change the entire treatment pathway (staging, systemic therapy, surgical approach), then the threshold to escalate should be lower than in a low-impact benign-leaning scenario. The published literature on these cases is likewise composed mainly of retrospective institutional series, so conclusions should be interpreted in the context of referral pattern and underlying case mix [51, 52].

Overall, the direction of evidence across multiple studies is consistent: optimized sampling, ancillary-ready processing, and selective escalation to tissue acquisition increase the likelihood that preoperative diagnosis will be clinically actionable [19, 23–25, 36, 37, 40]. However, interpretation should consider the narrative nature of this review and the lack of formal meta-analytic pooled estimates for individual workflow components, which limits quantitative comparisons across strategies; moreover, some special diagnostic scenarios are necessarily informed by relatively small retrospective institutional series [49–52]. Future research should therefore prioritize pathway-level outcomes under Milan System 2nd edition implementation, including non-diagnostic rate, specific diagnosis rate, re-biopsy rate, time to definitive management, and complication rates across different resource settings. A second priority is harmonization across sampling modalities, including validation of Milan-like risk stratification for core needle biopsy to support consistent ROM/RON communication within integrated diagnostic pathways [40, 41].

## Conclusion

A preoperative pathway guided by the Milan System 2nd edition is best framed as an integrated, multidisciplinary diagnostic workflow coordinating cytology with clinical and imaging findings, rather than as a decision based on a single test result. Ultrasound-guided FNA remains the optimal first-line approach when supported by ROSE/tele-ROSE and consistent preparation of ancillary-ready cell-blocks [2, 6, 8, 19, 20, 23]. Ancillary testing (IHC/FISH/NGS) then becomes a predictable capability for resolving indeterminate and high-impact cases [24, 33]. CNB complements this strategy as targeted escalation when architecture, tissue volume, robust subtyping, grading, or extensive ancillary profiling is essential, supported by comparative and meta-analytic evidence for higher sensitivity and lower non-diagnostic rates than FNA with generally rare complications [36, 38, 39]. By integrating these elements, the pathway reduces gray-zone reporting, increases clinically actionable diagnoses, and aligns salivary cytopathology with contemporary expectations of multidisciplinary, precision-informed care.

## Data Availability

The word file and pictures of the different entities are available upon request.
